# Supplementary material for: 20%-efficient polycrystalline Cd(Se,Te) thin-film solar cells with compositional gradient near the front junction
Source: Nat Commun. 2022 Dec 21;13:7849. doi: 10.1038/s41467-022-35442-8 (PMC9772316; doi:10.1038/s41467-022-35442-8)
Supplement: Supplementary file 1 — Supplementary Information [file 41467_2022_35442_MOESM1_ESM.pdf]

Supplementary Information

20%-efficient Polycrystalline Cd(Se,Te) Thin-Film Solar Cells with Compositional Gradient near the Front Junction

Li et al.

Supplementary Table 1. Parameters used for SCAPS 1D device simulation.

Parameters	SnO_2	Cd(S,Se,Te)	Cd(Se,Te)	CdTe	CuSCN
Thickness (um)	0.30	0.10	1.00	2.00	0.10
Bandgap (eV)	3.60	1.50	1.38	1.50	3.70
Electron affinity (eV)	4.45	4.40	4.45	4.40	2.00
N_{CB} (1/cm^3)	2.20E18	8.00E17	8.00E17	8.00E17	8.00E17
N_{VB} (1/cm^3)	1.80E19	1.80E19	1.80E19	1.80E19	1.80E19
Electron thermal velocity (cm/s)	1.00E7	1.00E7	1.00E7	1.00E7	1.00E7
Hole thermal velocity	1.00E7	1.00E7	1.00E7	1.00E7	1.00E7
Electron mobility (cm^2/Vs	50	3.20E2	3.20E2	320	320
Hole mobility (cm^2/Vs)	25	40	40	40	40
N_A (1/cm^3)		2.00E14	5.00E14	2.00E15	2.00E16
N_D (1/cm^3)	5E20				
Tau e/h		300/300	300/300	59/59	1.50E-2/1.50E-2
Interface trap state	N_A:6.00E13, E_t=0.60 eV above middle of IF gap, capture cross section for electron:5.00E-12cm^2, hole: 5.00E-12cm^2				

Note: In the contour simulation, the bandgap of Cd(S,Se,Te) layer is varied between 1.5 to 2.0 eV and the trap state density at the SnO_2/Cd(S,Se,Te) interface is varied between 1.00E10^{12} to 1.00E10^{16}/cm^3. For the convergence of all the data point, the electron affinity of Cd(S,Se,Te) is set to be 4.35 eV in the contour simulation.

Supplementary Table 2. The values of V_{oc}^{rad} (the ideal V_{OC} determined by the band edge radiative recombination), V_{loss} (the open circuit voltage loss due to non-radiative recombination), and iV_{OC} (implied voltage or internal voltage determined by the difference between the V_{oc}^{rad} and V_{loss})

	V_{oc}^{rad} (mV)	PLQY (%)	iV_{oc} (mV)	V_{oc}^{loss} (mV)
Control	1160 ± 10	9.0e-4	858 ± 13.5	302 ± 3.5
Target	1161 ± 10	3.4e-3	893 ± 12.2	266 ± 2.2

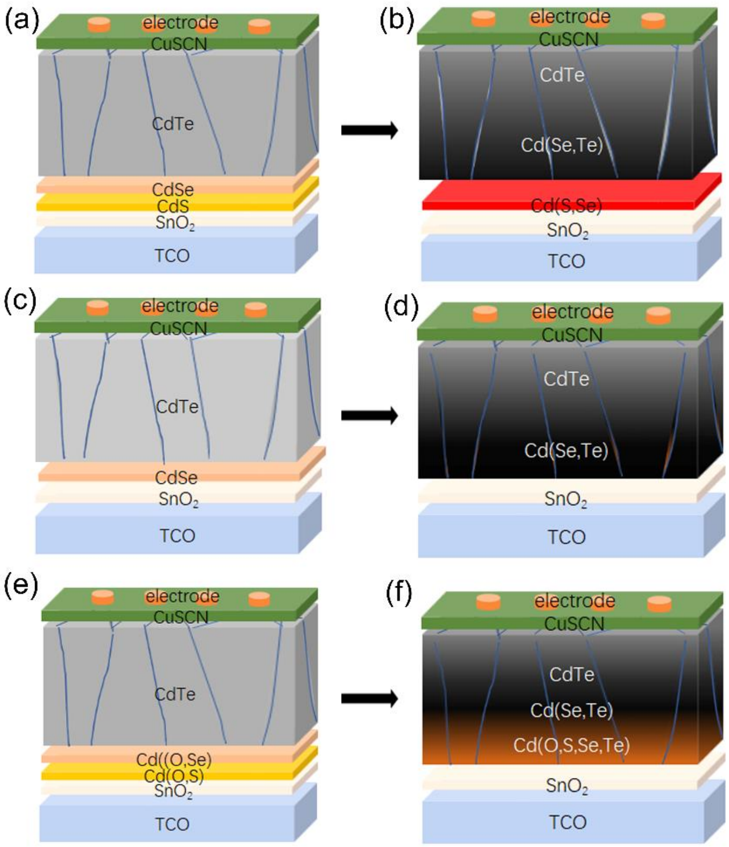

Supplementary Figure 1. Illustrations of film stacks before (a) CdS/CdSe/CdTe layers, (c) CdSe/CdTe layers, and (e) Cd(O,S)/Cd(O,Se)/CdTe layers and after ((b), (d), (f) $CdCl_2$ heat treatment.

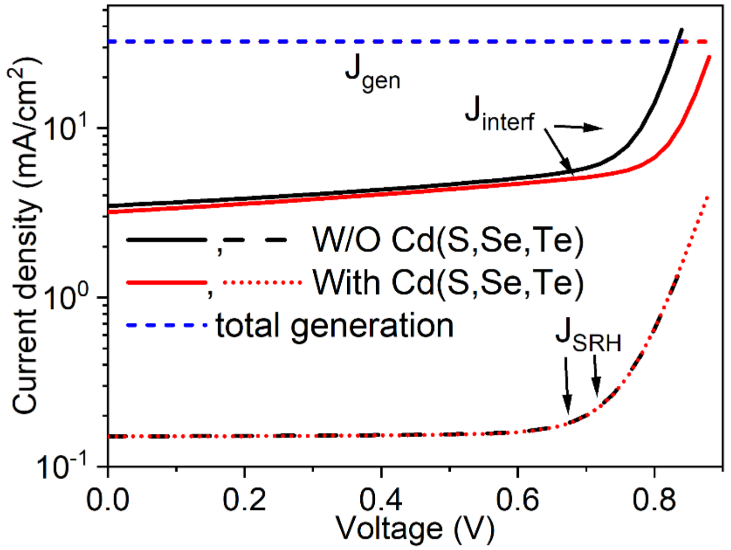

Supplementary Figure 2. SCAPS simulated bias-dependent recombination current density and for devices with and without the bandgap gradient at the front interface.

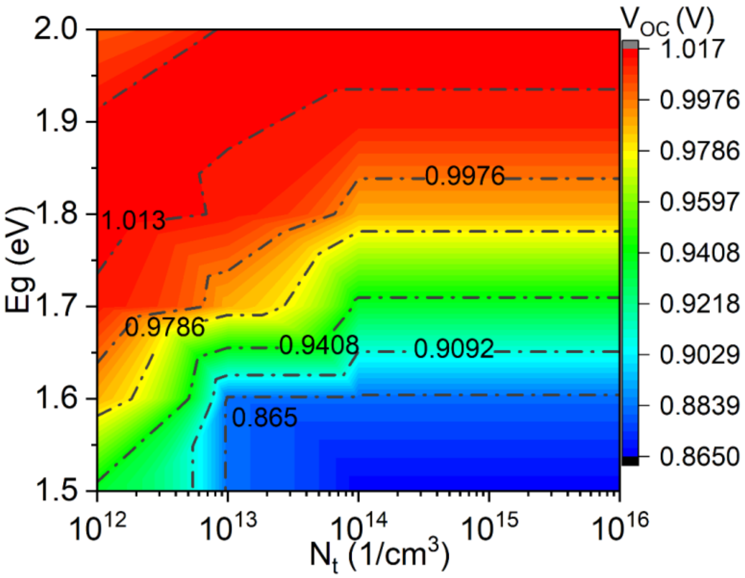

Supplementary Figure 3. SCAPS simulated contour plot of the V_{OC} of devices with ranges of trap density at SnO_2/Cd(S,Se,Te) interface and the bandgap of the Cd(S,Se,Te) region. The parameters used in the SCAPS simulations are provided in Supplementary Table 1.

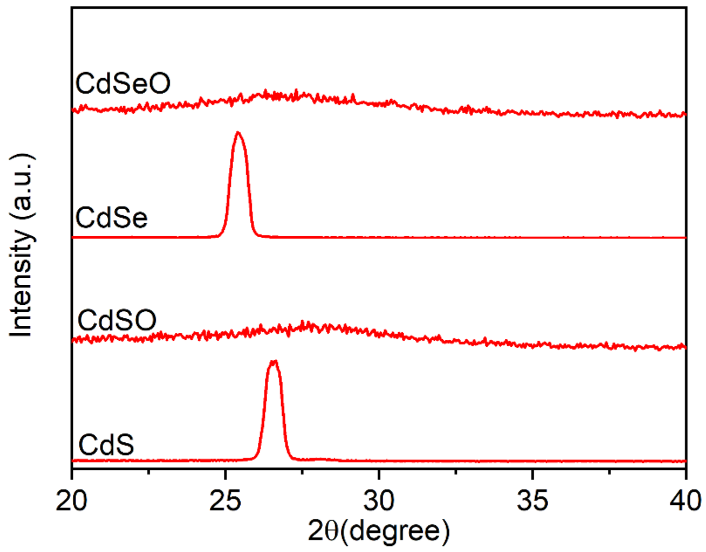

Supplementary Figure 4. X-ray diffraction patterns for the CdS and CdSe films deposited with and without the presence of oxygen during sputtering.

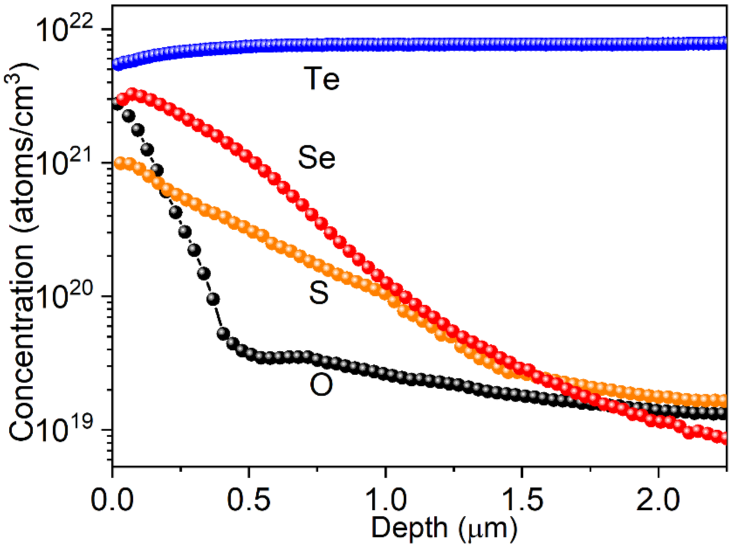

Supplementary Figure 5. Time-of-flight secondary ion mass spectrometry (TOF SIMS) depth profiles for a delaminated Cd(Se,Te) device fabricated with Cd(O,S) and Cd(O,Se). The left y-axis on the SIMS depth profile indicates the location of the absorber/SnO_2 front interface.

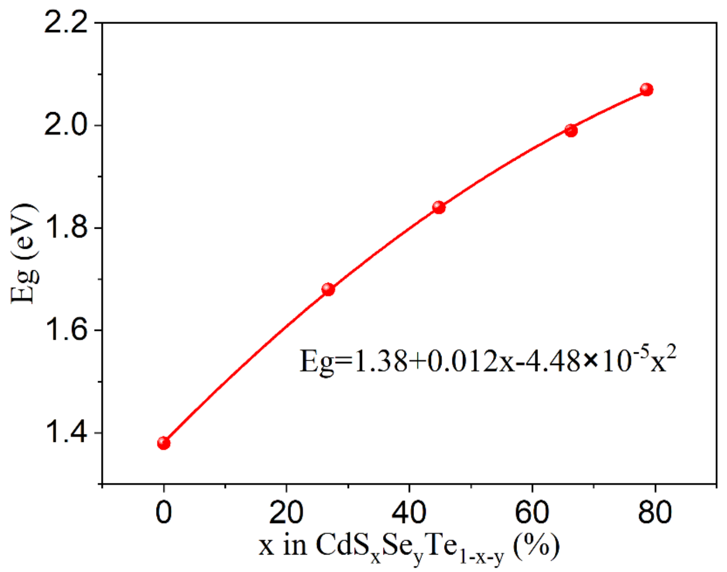

Supplementary Figure 6. Polynomial fitting (polynomial order is 2) of the bandgaps of evaporated Cd(S,Se,Te) ($CdS_xSe_yTe_{1-x-y}$) films with different x values. For each Cd(S,Se,Te) film, the deposition rates of CdSe and CdTe were fixed to obtain a Se/(Se+Te) ratio of 38%, and the S composition was regulated by changing the CdS deposition rate.

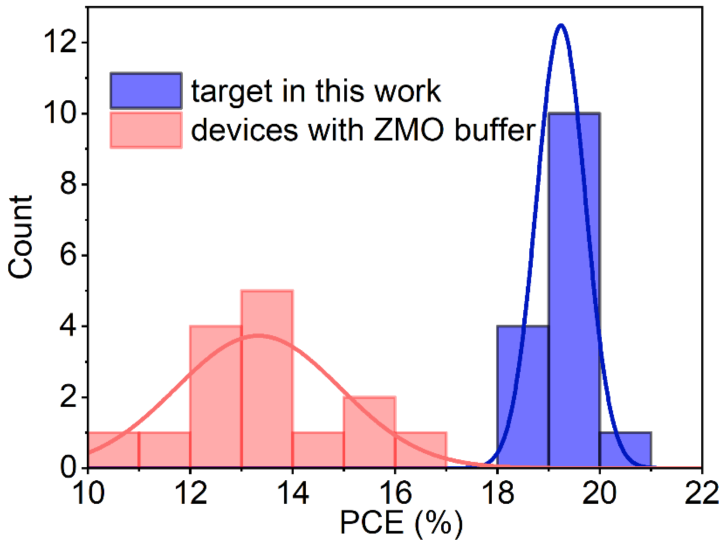

Supplementary Figure 7. Comparison of reproducibility of devices. Statistics of device performance for the target cells and cells using ZMO as buffers (~15 cells for each type)

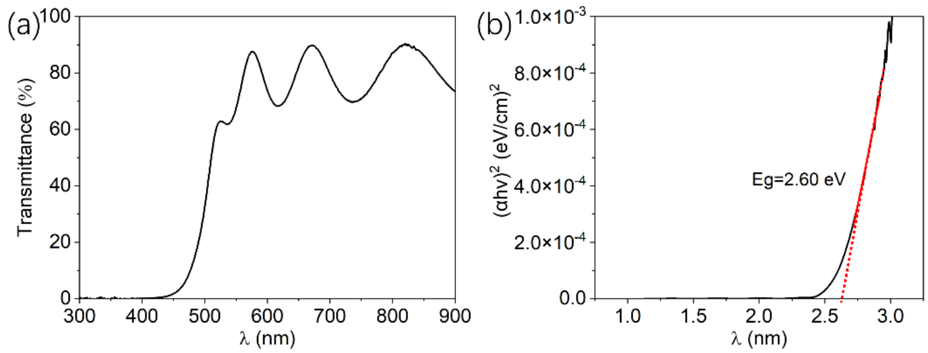

Supplementary Figure 8. (a) Transmittance spectrum and (b) tauc plot (n=2, direct) of a Cd(O,S) film deposited in 2% oxygen and 98% argon on soda-lime glass.

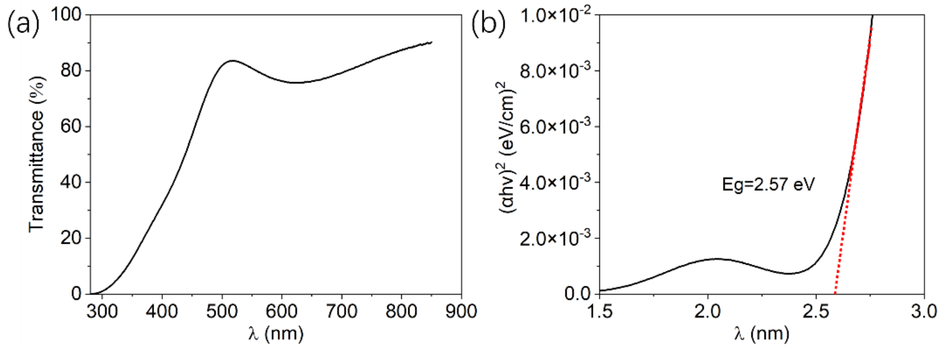

Supplementary Figure 9. (a) Transmittance spectrum and (b) tauc plot (n=2, direct) of a Cd(O,Se) film deposited in 2% oxygen and 98% argon on soda-lime glass.
