# Supplementary material for: 20%-efficient polycrystalline Cd(Se,Te) thin-film solar cells with compositional gradient near the front junction
Source: Nat Commun. 2022 Dec 21;13:7849. doi: 10.1038/s41467-022-35442-8 (PMC9772316; doi:10.1038/s41467-022-35442-8)
Supplement: Supplementary file 3 — Solar Cells Reporting Summary [file 41467_2022_35442_MOESM3_ESM.pdf]

nature research

Corresponding author(s): Yanfa Yan

Solar Cells Reporting Summary

Nature Research wishes to improve the reproducibility of the work that we publish. This form is intended for publication with all accepted papers reporting the characterization of photovoltaic devices and provides structure for consistency and transparency in reporting. Some list items might not apply to an individual manuscript, but all fields must be completed for clarity.

For further information on Nature Research policies, including our data availability policy, see Authors & Referees.

Experimental design

Please check: are the following details reported in the manuscript?

1. Dimensions

Area of the tested solar cells

- [x] Yes — Methods - Device fabrication. Cells area of 0.08 cm2
- [ ] No — *Explain why this information is not reported/not relevant.*

Method used to determine the device area

- [x] Yes — Methods - Device fabrication. The active area was determined by the mask used for the gold electrode deposition.
- [ ] No — *Explain why this information is not reported/not relevant.*

2. Current-voltage characterization

Current density-voltage (J-V) plots in both forward and backward direction

- [ ] Yes — *State where this information can be found in the text.*
- [x] No — Hysteresis is not oberved

Voltage scan conditions
For instance: scan direction, speed, dwell times

- [x] Yes — Methods - Characterization of the CdTe device. From -0.3 V to 1.3 V bias with a scan speed of 200 mV/s
- [ ] No — *Explain why this information is not reported/not relevant.*

Test environment
For instance: characterization temperature, in air or in glove box

- [x] Yes — Methods - Characterization of the CdTe device. The cells were measured at room temperature in air.
- [ ] No — *Explain why this information is not reported/not relevant.*

Protocol for preconditioning of the device before its characterization

- [x] Yes — Before J-V measurement, light soaking treatment was carried out for all the devices at 85°C under AM1.5G illumination for 15 min.
- [ ] No — *Explain why this information is not reported/not relevant.*

Stability of the J-V characteristic
Verified with time evolution of the maximum power point or with the photocurrent at maximum power point; see ref. 7 for details.

- [ ] Yes — *State where this information can be found in the text.*
- [x] No — Stability testing is beyond the scope of this article.

3. Hysteresis or any other unusual behaviour

Description of the unusual behaviour observed during the characterization

- [ ] Yes — *State where this information can be found in the text.*
- [x] No — Hysteresis is not observed.

Related experimental data

- [ ] Yes — *State where this information can be found in the text.*
- [x] No — Hysteresis is not observed.

4. Efficiency

External quantum efficiency (EQE) or incident photons to current efficiency (IPCE)

- [x] Yes — Figure 4f
- [ ] No — *Explain why this information is not reported/not relevant.*

A comparison between the integrated response under the standard reference spectrum and the response measure under the simulator

- [x] Yes — methods
- [ ] No — *Explain why this information is not reported/not relevant.*

For tandem solar cells, the bias illumination and bias voltage used for each subcell

- [ ] Yes — *State where this information can be found in the text.*
- [x] No — These are not tandem cells

5. Calibration

Light source and reference cell or sensor used for the characterization

☒ Yes ☐ No

Methods - Characterization of the CdTe device. The light intensity for J–V measurements was calibrated by a standard silicon wafer solar cell certified by Newport. A standard silicon wafer solar cell was used as the reference for the EQE measurement.

Explain why this information is not reported/not relevant.

Confirmation that the reference cell was calibrated and certified

☒ Yes ☐ No

Methods - Characterization of the CdTe device. The light intensity for J–V measurements was calibrated by a standard silicon wafer solar cell certified by Newport. A standard silicon wafer solar cell was used as the reference for the EQE measurement.

Explain why this information is not reported/not relevant.

Calculation of spectral mismatch between the reference cell and the devices under test

☒ Yes ☐ No

Methods - Characterization of the CdTe device. A standard silicon wafer solar cell was used as the reference for the EQE measurement.

Explain why this information is not reported/not relevant.

6. Mask/aperture

Size of the mask/aperture used during testing

☐ Yes ☒ No

State where this information can be found in the text.

the Au electrode area of the cells was measured directly

Variation of the measured short-circuit current density with the mask/aperture area

☐ Yes ☒ No

State where this information can be found in the text.

the Au electrode area of the cells was measured directly

7. Performance certification

Identity of the independent certification laboratory that confirmed the photovoltaic performance

☐ Yes ☒ No

State where this information can be found in the text.

The light intensity of our solar simulator for J-V and EQE measurement was calibrated by a standard silicon wafer solar cell

A copy of any certificate(s)
Provide in Supplementary Information

☐ Yes ☒ No

State where this information can be found in the text.

The light intensity of our solar simulator for J-V and EQE measurement was calibrated by a standard silicon wafer solar cell

8. Statistics

Number of solar cells tested

☒ Yes ☐ No

Figure 4 caption; Supplementary Fig. 5 caption. 15 cells for each device

Explain why this information is not reported/not relevant.

Statistical analysis of the device performance

☒ Yes ☐ No

Figure 4a-d; Supplementary Fig. 5

Explain why this information is not reported/not relevant.

9. Long-term stability analysis

Type of analysis, bias conditions and environmental conditions
For instance: illumination type, temperature, atmosphere humidity, encapsulation method, preconditioning temperature

☐ Yes ☒ No

State where this information can be found in the text.

Long term stability testing is beyond the scope of this article.
